# Evaluation of a Healthy Relationship Smartphone App With Indigenous Young People: Protocol for a Co-designed Stepped Wedge Randomized Trial

**DOI:** 10.2196/24792

**Published:** 2021-12-30

**Authors:** Jane Koziol-McLain, Denise Wilson, Alain C Vandal, Moana Eruera, Shyamala Nada-Raja, Terry Dobbs, Michael Roguski, Te Wai Barbarich-Unasa

**Affiliations:** 1 Centre for Interdisciplinary Trauma Research Auckland University of Technology Auckland New Zealand; 2 Taupua Waiora Centre for Māori Health Research Auckland University of Technology Auckland New Zealand; 3 Department of Statistics University of Auckland Auckland New Zealand; 4 Oranga Tamariki Ministry for Children Wellington New Zealand; 5 Centre for Pacific Health University of Otago Dunedin New Zealand; 6 Kaitiaki Research and Evaluation Wellington New Zealand

**Keywords:** indigenous, Māori, young people, relationships, school, mHealth, smartphone app, mobile phone

## Abstract

**Background:**

We co-designed a smartphone app, Harmonised, with taitamariki (young people aged 13-17 years) to promote healthy intimate partner relationships. The app also provides a pathway for friends and family, or whānau (indigenous Māori extended family networks), to learn how to offer better support to taitamariki.

**Objective:**

The aim of our taitamariki- and Māori-centered study is to evaluate the implementation of the app in secondary schools. The study tests the effectiveness of the app in promoting taitamariki partner relationship self-efficacy (primary outcome).

**Methods:**

We co-designed a pragmatic, randomized, stepped wedge trial (retrospectively registered on September 12, 2019) for 8 Aotearoa, New Zealand, secondary schools (years 9 through 13). The schools were randomly assigned to implement the app in 1 of the 2 school terms. A well-established evaluation framework (RE-AIM [Reach, Effectiveness, Adoption, Implementation, Maintenance]) guided the selection of mixed data collection methods. Our target sample size is 600 taitamariki enrolled across the 8 schools. Taitamariki will participate by completing 5 web-based surveys over a 15-month trial period. Taitamariki partner relationship self-efficacy (primary outcome) and well-being, general health, cybersafety management, and connectedness (secondary outcomes) will be assessed with each survey. The general effectiveness hypotheses will be tested by using a linear mixed model with nested participant, year-group, and school random effects. The primary analysis will also include testing effectiveness in the Māori subgroup.

**Results:**

The study was funded by the New Zealand Ministry of Business, Innovation, and Employment in October 2015 and approved by the Auckland University of Technology Ethics Committee on May 3, 2017 (application number: 17/71).

**Conclusions:**

This study will generate robust evidence evaluating the impact of introducing a healthy relationship app in secondary schools on taitamariki partner relationship self-efficacy, well-being, general health, cybersafety management, and connectedness. This taitamariki- and indigenous Māori–centered research fills an important gap in developing and testing strengths-based mobile health interventions in secondary schools.

**Trial Registration:**

Australian New Zealand Clinical Trials Registry ACTRN12619001262190; https://www.anzctr.org.au/Trial/Registration/TrialReview.aspx?id=377584

**International Registered Report Identifier (IRRID):**

RR1-10.2196/24792

## Introduction

### Study Rationale

Abuse in intimate partner relationships is a human rights violation and a social and public health problem [1,2]. In Aotearoa, New Zealand, 1 in 3 women experience physical or sexual violence by a partner, with rates higher for indigenous Māori (58%) compared with New Zealand European (34%) women [3,4]. For many, their first experience of relationship abuse is during adolescence (13-18 years) [5-9]. Relationship abuse may include psychological, physical, sexual, or cyber abuse threats [10-12]. There is a small but growing body of literature examining relationship abuse prevalence, prevention, and intervention during this critical period of adolescence, when individuals transition from childhood to adulthood [13-15]. Comprehensive, accessible, innovative, and cost-effective interventions are required to prevent intimate partner abuse among young people. However, frequently, research programs appear to be done *on* rather than *with* young people, and few studies provide an indigenous lens. Given the significant health and social inequities of indigenous Māori in Aotearoa, New Zealand, we embarked on a program of co-designed research to develop and evaluate a personalized healthy relationship smartphone app using a *taitamariki*- (young person) and Māori-centered approach. The RE-AIM (Reach, Effectiveness, Adoption, Implementation, Maintenance) framework, including qualitative and quantitative data collection methods, was used to examine the adoption and implementation of the app in 8 pilot secondary schools in New Zealand [16,17]. In this paper, we present a trial protocol for testing the effectiveness of the app.

### Taitamariki-Centered Approach

As a team, we are committed to including the voice of young people in our work, consistent with their right to express opinions freely and have these considered in any matter that affects them [18,19]. We will convene *taitamariki* advisory groups (TAGs) to enable young people to cocreate the app using participatory research principles [20] and cocreation processes [21,22]. Before the formal trial, Māori team members experienced in child advocacy and focus group methodology (ME, TD, and TWBU) recruited and facilitated 2 cohorts of TAGs. TAG members and their parents provided written consent to participate in this research project. The initial *tuakana* (older) TAG included 7 *taitamariki* from 1 New Zealand region. At recruitment, they ranged in age from 15 to 17 years and included both *taitamatane* (boys; n=4) and *taitamahine* (girls; n=3); 5 of the 7 *taitamariki* self-identified as Māori. As the app was developed and trial was planned, meetings were convened *kanohi-ki-te-kanohi* (face-to-face), supplemented with communication via a private Facebook group. The second *teina* (younger) TAG members are recruited from our pilot schools across New Zealand and include 15 *taitamariki* (boys, n=4; girls, n=11). Communication includes *kanohi-ki-te-kanohi* meetings, videoconferencing, and interaction on Instagram. TAG members participated in project branding, app development, and trial design. The project branding *Harmonised* was chosen by the TAG. The TAG members will participate in the implementation, interpretation of findings, and dissemination.

### Māori-Centered Approach

As a team, together with our community advisors, we are committed to a Māori-centered approach. At our first team *hui* (meeting), we specified our purpose, *kaingākau* (values), *tikanga* (right way of doing things in a Māori worldview), and *whanonga* *pono* (principles) to guide our *mahi* (work). A Māori-centered approach is premised on the bicultural relationship between Māori as *tangata* *whenua* (indigenous people of the land) and *tauiwi* (nonindigenous people) [23]. This research privileges a Māori worldview, ensuring that processes and outcomes are beneficial for Māori, inclusive of Māori values, expectations, and needs, and cedes control to protect Māori interests.

Early in the development of this research program, we took a philosophical turn from the mainstream deficit-based approach of reducing violence to a Māori well-being and strengths-based approach of promoting healthy relationships. From that point, we problematized our processes and decisions. For example, we identified that available validated measures focused on measuring adolescent relationship abuse (or *dating violence*) rather than measuring healthy relationships—the core focus of the research. In addition, identified measures were predominantly developed for adults and *modified* for young people, often without input from young people themselves. Finally, we could identify no measures for our variables of interest that represented a Māori or indigenous youth perspective. Therefore, in many respects, we are traveling uncharted territory, balancing cross-disciplinary and cross-cultural bodies of knowledge.

### Why Mobile Health?

As people are increasingly seeking health information from the internet [24,25], mobile health interventions offer promise for improved health and well-being across the life span [26,27]. There is also evidence of value in the development of mobile health interventions aligned with indigenous frameworks [28-30]. In research with *taitamariki* Māori in Northland New Zealand about healthy and unhealthy *intimate relationships*, *taitamariki* expressed a need for more information and more effective support from friends and whānau [31,32]. However, rather than being *lectured to or given advice when they haven’t asked for it*, they suggested *safe, easily accessible social media and web-based tools to use privately* [33]. The Harmonised app is meant to address this information need identified by *taitamariki* Māori. The intent is to develop a resource that would supplement, rather than replace, other healthy relationship learning in schools, whānau, or families or communities. New Zealand secondary schools have a range of digital technology devices [34] and a Ministry of Education–funded Chrome Education License [35].

### Harmonised Healthy Relationship App

**Figure 1:**
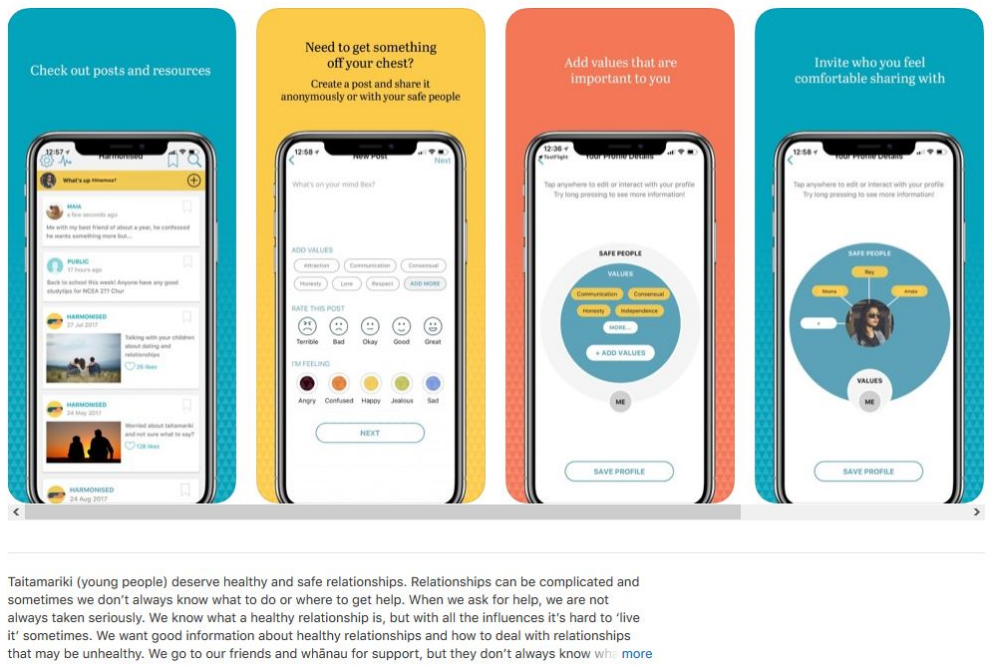
Screenshot from the Apple App Store (version 1.8.3; August 2019).

The Harmonised healthy relationship smartphone app was developed consistent with our *taitamariki*- and Māori-centered approach. Development was iterative, involving repeated cycles of *taitamariki* input, app development, and testing. Our software engineer and app developer worked together in applying the specifications identified by *taitamariki* in focus groups, usability testing, and by our TAG members (Figure 1). *Taitamariki* specified their desire for an interactive, private social network that allows *taitamariki* users to (1) create their own relationship profile and choose their relationship values, (2) learn about common relationship issues (healthy relationship information and skill resources), and (3) post issues or comments about their relationships with others. *Taitamariki* control whether to make posts private (sharing only with selected *safe people*) or public (shared anonymously). Responding to the *taitamariki* desire for better support from their friends and whānau, the Harmonised app supports 2 user types: (1) primary users are *taitamariki*, with access to all app functions, and (2) secondary users are friends, whānau, or family and community who have access to healthy relationship information resources only, unless invited as a primary user’s *safe person* to comment on a specific post.

A Harmonised moderation protocol guides safe practices for all posts (posts and comments), minimizing the risk of harm to primary app users, harmful use of the app, and harm to the platform. The protocol is aligned with best practice safety and security measures from Netsafe [36], Technology Safety [37], and others [21,38]. The moderation protocol includes automated (inappropriate language block and suicidality or self-harm automated message), human (daily review by the research team with the ability to block posted comments or individual users), community-based (users’ report inappropriate content), and reputation-based moderation. An escalation pathway identifies procedures, should moderators have a safety concern. The safe and responsible use of the app is communicated to all users. The protocol is aligned to the New Zealand Harmful Digital Communications Act 2015.

Healthy relationship resources are collated by a Harmonised resource working group to provide *taitamariki* with the information they have requested. The topics covered include, for example, communication, consent, dealing with anger, and how to talk with your parents or children about healthy relationships and sexuality. The working group followed a protocol for resource selection, modification, and permission for use. Resources include links to videos, quizzes, brief articles, and stories from a range of open-access sources. Where to get help is a *recommended* resource for all users. New Zealand and Māori resources were prioritized.

Following a beta test version available in June 2017, version updates continued with bug *fixes* and enhancements. In some cases, changes were made in response to requests by school staff (eg, ability for persons to accept or not accept a *safe person’s* invitation). As a prevention intervention, there is also attention to promoting the app for all *taitamariki*, regardless of relationship status. For example, presenting the interactive selection of relationship values as a requirement before advancing to the app *feed* allows all *taitamariki* to participate in the healthy relationship values exercise. We also use gender-diverse language, sensitive to the minority (estimated at 3.8%) of Aotearoa young people attracted to others of the same sex or both sexes [21,39,40].

The hybrid app in English and Te Reo Māori (translation by a certified Māori language interpreter) is available across the digital ecosystem, including mobile devices (smartphones and tablets), laptops, and desktop computers. The app was first published in June 2018 in the Apple App Store, Google Play Store, and web browser by the Auckland University of Technology Enterprises Ltd. To increase privacy, the app is personal identification number code–protected to keep information safe once a user moves away from the screen. To reduce the risk of harm from *nontaitamariki* inappropriately posting (eg, trolling), the posting function is limited to students enrolled at participating schools and validated during app registration against email lists provided by the schools.

## Methods

### Trial Purpose and Hypotheses

We are investigating the adoption and implementation of the interactive, personalized healthy relationship Harmonised app in 8 pilot secondary schools in New Zealand using the well-established RE-AIM evaluation framework [16,17]. Mixed method data sources include a quantitative web-based *taitamariki* survey, focus groups with *taitamariki* and school stakeholders (persons identified by schools who are involved in their response to *taitamariki* health and well-being), app use data, school engagement notes, and itemized costs (Table 1). In this paper, we present the trial protocol for testing the effectiveness of the Harmonised app using a quantitative web-based *taitamariki* survey.

We hypothesize (primary analysis) that implementation of the app (compared with before implementation) will (1) increase *taitamariki* relationship self-efficacy (RSE; primary outcome) and (2) increase *taitamariki* well-being, general health, cybersafety management, and connectedness (secondary outcomes). We will also conduct (3) primary analyses limited to Māori participants (subgroup analyses). Other measures include school and student characteristics and *taitamariki* help-seeking and barriers to getting help.

**Table 1:** Harmonised evaluation data sources guided by the Reach, Effectiveness, Adoption, Implementation, and Maintenance framework.

Implementation	Quantitative data sources	Qualitative data sources
Engage with schools to adopt the Harmonised app	Number of schools agreeing to participate, number of schools invited, and proportion of invited schools agreeing to participate	Drivers of and barriers to school participation: School engagement log School engagement notes Preimplementation school information forms Preimplementation focus groups
The app is implemented in schools	Number of schools implementing in accordance with random assignment Costs of implementation (time and money)	Understanding implementation: School implementation plans Implementation run sheets Postimplementation stakeholder focus groups Postimplementation taitamariki focus groups
The app reaches the target population	Number and proportion of students in participating schools and others that download and use the app: App download data (Firebase reports) App use assessed in taitamariki survey postimplementation	Drivers of and barriers to app access and use: Open-ended query in survey reason for not downloading Postimplementation focus groups with taitamariki Postimplementation focus groups with stakeholders Whānau interviews
The app is effective at improving relationship and well-being outcomes	Primary and secondary outcomes assessed in web-based survey completed by taitamariki at 5 school terms over 18 months	Impacts of the app: Postimplementation focus groups with taitamariki
Maintenance of the Harmonised app beyond the implementation period	App use data posttrial (Firebase)	Understanding long-term impacts and app retention: Postimplementation hui (meeting)

### Trial Design

A pragmatic, stepped wedge, cluster randomized (one-directional crossover) trial in 8 pilot secondary schools tests the effectiveness of the Harmonised app. Secondary schools in New Zealand include years 9 through 13 (5 years of high school, generally young people aged 13 to 17 years). In the stepped wedge design, the app is implemented in all 8 schools in one of 2 school terms, with the order of implementation determined at random [41-44]. There are 5 assessments (web-based surveys) per school and 2 time steps over a 15-month trial period (Table 2). Our protocol description follows the CONSORT (Consolidated Standards of Reporting Trials) stepped wedge, cluster randomized trial extension [44]. The study was registered with the Australian New Zealand Clinical Trials Registry (ACTRN12619001262190) on September 12, 2019.

**Table 2:** Stepped wedge implementation design plan.

School ID Number	Year and school term					
	Year 1					Year 2, T1^a^
	T1^a^	T2^a^	T3^a^	T4^a^		
8	CC^b^	CC	TP^c^	IC^d^	IC	
7	CC	CC	TP	IC	IC	
6	CC	CC	TP	IC	IC	
5	CC	CC	TP	IC	IC	
4	CC	TP	IC	IC	IC	
3	CC	TP	IC	IC	IC	
2	CC	TP	IC	IC	IC	
1	CC	TP	IC	IC	IC	

^a^T1 to T4 are the terms for the implementation of the design plan.

^b^CC: control condition.

^c^TP: transition period.

^d^IC: intervention condition.

### Eligibility for Clusters: School Selection

#### Overview

The New Zealand Ministry of Education website provides school characteristics, including type of school, gender, ethnicity proportion, and other characteristics [45]. The 366 state schools in New Zealand include a mix of coeducational and boys- or girls-only schools across years 9 to 13. Although there are National Certificate of Educational Achievement unit standards for healthy relationships, their integration into secondary school curricula varies. Our inclusion criteria includes secondary or composite schools that had a school roll >175 (based on average class sizes for each year) and ≥16% Māori (proportion of Māori in the population; StatsNZ). This left 194 eligible schools. In this *taitamariki*- and Māori-centered study, we added 6 schools to the eligibility list that had a high proportion of Māori students and had participated in earlier *kaupapa* Māori research in the Ngāpuhi tribal boundaries within the Northland region of New Zealand [33].

#### School Recruitment

From the list of eligible schools, we will purposefully invite schools until we achieve our desired sample of 8 schools. We aim to balance girls-only, boys-only, and coeducational schools. We also aim for a balance of schools that have and have not participated in specialized healthy relationship curricula such as Mates and Dates (Accident Compensation Corporation) that are being piloted [46,47]. Finally, we will consider the geographic diversity and travel feasibility.

#### Implementation Conditions

The intervention consists of the implementation of the Harmonised app in each participating secondary school. An app implementation plan is being developed jointly with each school following a stakeholder focus group. A one-off single-period (40-50 minutes) implementation delivery occurs within individual classrooms or during school assembly. Participating schools may elect for app implementation sessions to be school-led or researcher-led. A hard-copy training resource designed specifically for the study supports implementation and includes instructions regarding app navigation and function. Researcher-led training includes demonstrations of app functions. Students are led through the process of registration, creation of user profiles, and creation of posts. Strategies to support app adoption include the use of posters and school newsletters. The intended frequency and duration of use of the app beyond the initial guided implementation is at the students’ discretion. The tailored nature of the implementation limits the evaluation of fidelity.

#### Comparison Condition

The comparison period includes school terms before app implementation from the clustered one-directional crossover design. In the preimplementation comparison period, researchers engage with an identified school liaison, convene a preimplementation focus group, and confirm the school’s pathway for referral of students needing health and well-being services, including social workers, counselors, and health providers. No control period activities that affect school students will be undertaken.

### Survey

#### Eligibility for Survey Participants: Student Selection Within Schools

Student eligibility criteria includes being enrolled in one of the pilot schools as a year 9-13 student and able to consent and complete a web-based survey. For schools with a roll <100, all students of years 9-13 are eligible to participate. For schools with a larger roll, all students in 1 class in a given subject for each year (9-13) are selected. Class selection is negotiated with the school liaison and prioritizes the inclusion of Māori students. A new class of year 9 (aged 13 years) is selected in each school in year 2, term 1 of the study.

#### Student Recruitment and Retention for the School Survey

We will attend selected classrooms during class time and invite eligible students to participate in the research by completing 5 web-based surveys over 5 school terms. Students are asked to share their understanding of what is being asked of them. During data collection sessions, there are discreet options available for students choosing not to participate. Consenting students complete the baseline survey on school digital devices during class time, with researchers available to respond should *taitamariki* have any questions as they complete the survey. Sandwiches are provided to all students at the end of class time. Students can exit the survey and complete it later. The remaining 4 surveys are completed independently by students in response to email and text nudges. Nudges are sent during week 5 of the subsequent 4 terms. Students are free to withdraw at any time by emailing the project. Survey data are exported into a secure server at the Auckland University of Technology.

#### Survey Development and Pilot-Testing

In preparation for the trial, the survey was pilot-tested in 2 schools (based on convenience) with approximately 60 students. This provided information on the time to complete the survey and identified the technical issues. Focus groups with students who completed the survey will be conducted to assess acceptability, comprehension, and appropriateness. Survey refinements will be made as indicated. Demographic characteristics include age, year in school, gender, ethnicity, internet exposure, and mobile phone access.

#### Outcomes

Students will complete web-based surveys at baseline (school term 1, year 1), 12 weeks (term 2, year 1), 24 weeks (term 3, year 1), 36 weeks (term 4, year 1), and 47 weeks (term 1, year 2). In selecting outcomes, the team considered the following: (1) positive, strengths-based measures (rather than deficit-based); (2) instruments developed with young people (rather than adult measures *modified* for young people); (3) instruments developed with indigenous young people; and (4) instruments developed with *taitamariki* Māori. At the time of study planning, there were no validated strengths-based measures developed with *taitamariki* Māori. The overwhelming majority of instruments used with young people measured *dating* or adolescent violence and had been modified from adult instruments.

Table 3 presents the final selection of the primary and secondary outcomes. All primary and secondary outcomes are assessed in each of the 5 surveys. Our primary outcome of interest is taitamariki RSE and includes 2 measures: confidence to talk about or seek help for themselves (RSE-self) and confidence to help others (RSE-others). The self-efficacy items are modeled on the Self-efficacy to Deal with Violence Scale [48,49]. In total, 2 items address RSE-self (How confident are you that you could check if parts of your relationship are ok, if you are not sure and How confident are you that you could seek help when your boyfriend or girlfriend has done something that’s not ok), and 2 items address RSE-others (How confident are you that you could help or support a friend or whānau member if they were not sure about parts of their relationships and How confident are you that you could help or support a friend or whānau member whose boyfriend or girlfriend has done something that’s not ok).

**Table 3:** Harmonised outcome measures.

Outcomes			Sources and modifications		Scales and subscales (number of items)		Response options		Possible score (range)
**Primary outcomes**									
	RSE^a^	Items modelled on Self-efficacy to Deal with Violence Scale [48,49]		RSE-self (2) RSE-others (2)		0=not at all confident 3=very confident		0-6 0-6	
**Secondary outcomes**									
	WB^b^	World Health Organization-Five Well-Being Index [50] used in children [51,52] and in New Zealand youth [53,54]. Modified “I have felt active and vigorous” to “I have felt active and full of energy”		WB (5)		0=at no time to 5=all the time		0-25	
	General health	Single 5-point Likert scale to rate respondent’s general health		General health (1)		0=poor to 4=excellent		0-4	
	Connectedness	Retained 2 subscales from Hemingway Measure of Adolescent Connectedness [55] with language regionalized and negatively worded items not scored		Connectedness-family or whānau (5) Connectedness-friends (5)		0=not at all true 4=very true		0-20 0-20	
	Cybersafety	15-item questionnaire began with a scenario modified from the Coping with Cyberbullying Questionnaire [56]; items are from original research with young Māori women [57]		Cybersafety–being safe (7) Cybersafety–taking action (8)		0=definitely not 3=definitely yes		0-21 0-24	

^a^RSE: relationship self-efficacy.

^b^WB: well-being.

The Cyber-Safety questionnaire was developed from original research on young Māori women [57]. The 15-item questionnaire begins with a scenario modified from the Coping with Cyberbullying Questionnaire [56], as follows:

Imagine that for a few weeks, you have been receiving nasty and threatening text messages. Aside from that, you found out that embarrassing pictures of you are being spread around.

Taitamariki then respond how likely they would be to use each of the 15 strategies to keep yourself safe on the internet (eg, I would talk to my friends about it, I pay attention to who has access to my data). Other secondary outcomes include well-being, general health, and connectedness (Table 3).

The baseline data (preimplementation of the intervention acquired in all schools before the transition period) will be extensively analyzed, leading to a full analytical design. In particular, exploratory factor analysis of the outcomes will lead to the creation or confirmation of subscores, making RSE, connectedness, and cybersafety bivariate outcomes.

#### Randomization

Schools are stratified by size (small or large). Large schools are further stratified by ongoing standardized delivery of a healthy relationship program (HRP). Within each stratum, the school labels are randomly ordered using a computer-generated sequence of pseudovariates. They are then assigned in this random order to the first sequence period (year 1, term 2: 2 small schools, 1 large school with HRP, and 1 large school with no HRP), and then the second sequence period (year 1, term 3: 1 small school, 2 large schools with HRP, and 1 large school with no HRP).

#### Analysis

The data will be kept stratified within the clusters by gender, Māori versus non-Māori ethnicity (hereafter identified as ethnicity), and year-group. The year-group (years 9-13) is defined as usual for the first 4 periods and crosses over to the next nominal year-group in the fifth period, so that each year-group defines a subcohort followed over time. The year 9 group from period 5 is identified as a separate year-group. The analysis sets consist of intention-to-treat, as-treated, and adopter (students reporting app use) sets. All primary analyses will take place in the intention-to-treat set. General effectiveness hypotheses will be tested using a linear mixed model with nested participant, year-group, and school random effects. All models will initially be fitted with the function *lme* from the R package *nlme* [58]. If the results fail to converge or otherwise display poor numerical behavior, PROC MIXED from SAS/STAT version 9.4 (SAS Institute Inc) will be used instead. 

To ensure the overlap of the intervention and control in the design, data will be collected during the transition periods and included in the analysis, assuming an intervention effect half the size of that in the posttransition periods. This approach is nonstandard but necessary in this instance and broadly plausible under the conditions of implementation and the nature of the intervention.

Māori subgroup analyses are planned. They will consist of all primary analyses limited to Māori participants. Subgroup analyses will take place in the intention-to-treat and as-treated sets. It will extend to all outcomes covered by primary analyses.

A blind review of the data will take place (before allocation unblinding) to determine whether any transformation is necessary, to settle on the final models, and to determine whether any missing covariate or outcome data require multiple imputation, and generally to finalize the statistical analysis plan. All tests will be performed at a 5% significance level against 2-sided alternatives. There are no circumstances in which unblinding is permissible.

#### Sample Size

Recruitment of 8 schools and data collection over 5 terms is judged feasible, with app implementation (the intervention) scheduled at 2 time points (terms 2 and 3, respectively, in the first year). We assumed roughly equal numbers of participants from each school. We use the method of Hussey and Hughes [59] to compute the power for different effect sizes under a model including the primary outcome (RSE), school-related random effect, and fixed effect associated with the term. The sample size computation was programmed in R version 3.x (R Foundation for Statistical Computing) by the study statistician in accordance with the analysis plan, including the specification that the intervention effect is assumed to be halved during the transition period. Other covariates may be included in the model, as decided during the blind review of the data.

Assuming an attrition of 35% (conservatively applied to all assessment time points postbaseline) and using a school-specific intraclass correlation of 0.07, evidenced in a bullying study in New Zealand schools [60], we estimate that recruiting 600 students is sufficient for detecting an effect size of 0.25 with 83% power and an effect size of 0.30 with 94% power. These correspond respectively and approximately to a change of 0.75 and 0.9 in the mean score of either component of the RSE score, based on the baseline data.

### Ethics and Safety

The trial protocol was approved by the Auckland University of Technology Ethics Committee (application number: 17/71), approved on May 3, 2017. All schools have a pathway for students needing health and well-being services, including social workers, counselors, and health providers. Consent to participate is provided by each school’s principal and Board of Trustees. Schools follow their processes for sharing information about the study with parents and gaining parental consent (information and consent forms provided by researchers). Students choosing to participate in the survey provide consent in the introduction to the web-based survey that details confidentiality. Students were identified using a randomized code number. All communication and visits with schools are documented by the research staff and reviewed by senior investigators as contextual data and to audit trial conduct. Our Harmonised ethical research practice and data sharing protocol provides a process for accessing Harmonised data aligned with our Māori-centered approach, available on request. Guided by our *tikanga*, we will prioritize *taitamariki*, schools, and whānau to disseminate our findings.

## Results

The study was funded by the New Zealand Ministry of Business, Innovation, and Employment in October 2015 and approved by the Auckland University of Technology Ethics Committee on May 3, 2017 (application number: 17/71). A total of 8 schools were recruited, and data were collected over 5 school terms.

## Discussion

### Principal Findings

The Harmonised trial will generate robust evidence to evaluate the impact of introducing a healthy relationship app into secondary schools. Importantly, this strengths-based *taitamariki*- and Māori-centered research counters the dominant adult-focused and deficit-based intimate partner violence literature. Working with *taitamariki* and community advisors, we have created the Harmonised brand focusing on what young people have told us about healthy partner relationships. The Harmonised app provides a safe digital network with resources for *taitamariki* to consider the values that are important to them for a healthy intimate partner relationship.

### Conclusions

This pragmatic trial offers an opportunity and challenge to understand whether a healthy relationship digital resource can be embedded in the secondary school environment and whether the resource benefits *taitamariki*, particularly Māori. Secondary schools are busy places that are typically underresourced to meet all the complex needs to support students and families to flourish. Our Harmonised study guided by explicit *tikanga* and using mixed methods guided by the RE-AIM framework will make an important contribution to understanding drivers of and barriers to conducting research in this unique setting. Our trial measures will identify whether the introduction of the app improves *taitamariki* partner RSE, well-being, general health, cybersafety management, and connectedness.

## Abbreviations

CONSORT: Consolidated Standards of Reporting Trials
HRP: healthy relationship program
RE-AIM: Reach, Effectiveness, Adoption, Implementation, Maintenance
RSE: relationship self-efficacy
TAG: taitamariki advisory group
